# Exploring the relationships between team leader’s conflict management styles and team passion: From the emotional perspective

**DOI:** 10.3389/fpsyg.2022.921300

**Published:** 2022-11-14

**Authors:** Jielin Yin, Meng Qu, Ganli Liao, Muxiao Jia, Miaomiao Li

**Affiliations:** School of Economics and Management, Beijing Information Science and Technology University, Beijing, China

**Keywords:** cooperative conflict management style, competitive conflict management style, positive emotional climate, team emotional intelligence, team passion

## Abstract

From the emotional perspective, this study explores how team leader’s conflict management styles affect team passion. A theoretical model is constructed which describes the mediating role of positive team emotional climate and the moderating impact of team emotional intelligence. We collect 101 teams paired data including 101 team leaders and 383 team members to test theoretical model. It is shown that leader’s cooperative conflict management style has a significant positive effect on both positive team emotional climate and team passion. Meanwhile, positive team emotional climate plays a mediating role between leader’s cooperative conflict management style and team passion. In addition, team emotional intelligence has a moderating effect between leader’s cooperative style and positive team emotional climate. This study not only provides a new perspective for follow-up research but also expands the research scope of impacts of conflict management styles. In addition, this study forms the underlying mechanism of team leader’s conflict management styles on team passion from the emotional perspective and investigates the mediating effect and moderating effect of emotional variable, which broadens the research on the mechanisms of conflict management styles on team outcomes to a certain extent.

## Introduction

Team work, which emphasizes collaboration and division of labor, is very effective in improving the flexibility of enterprises to cope with the ever-changing internal and external environment. Due to this, teams are widely used in organizations. In a team, the leader and members, for the most part, differ in their backgrounds, preferences, needs, standards, norms, values, and ethics, but they need to actively cooperate and interact to achieve organizational goals (García and Corbett, 2013). Therefore, conflicts within the team are everywhere and unavoidable. Conflict itself is harmless and its handling styles can greatly impact the team or the organization. If it is not properly handled, it may lead to the instability among teams or organizations in the short to long term. However, if it is properly handled, it can bring positive results to the long-term development of the team or the organization (Shih and Susanto, 2010; Way et al., 2016). Consequently, it seems studying conflict management is more worthy rather than conflict itself in this particular setting. Whether in organizational-level or team-level, conflicts occur either between employees or between employees and leaders. Raithel et al. (2021) propose that team leaders have a critical effect to deal with conflicts in the team. Therefore, team leader’s conflict management style (CMS) as one of the most important manifestations of leaders’ behaviors, which probably has a significant effect on team members’ attitudes and behaviors.

The existing literature on the influences of CMSs usually split them into two categories. One focuses on the impacts on team climate and team performance (Shih and Susanto, 2010; Tabassi et al., 2018; Wang et al., 2020), the other is concerned about the impacts on employees’ attitudes and behaviors (Erkutlu and Chafra, 2015; Leon-Perez et al., 2015; Way et al., 2016; Einarsen et al., 2018). Generally, most of related studies are based on cognitive rather than emotional perspective. With the cognitive perspective, previous studies mainly investigate the impacts of CMSs on individual and team outcomes based on social cognitive theory and social exchange theory such as employee innovation (Desivilya et al., 2010) and team coordination (Tabassi et al., 2018). However, a few scholars attempt to probe the relationships between conflict management styles and emotional related outcomes including collective emotional exhaustion (Benitez et al., 2018) and emotional intelligence (Al-Hamdan et al., 2019) with an emotional perspective. Yin et al. (2020b) believes that emotion is a very vital notion for a team. Since many human emotions are generated in interpersonal communication, emotion is critical to a team, especially as the internal mechanism that helps us understand team state and behavior (Chiang et al., 2020). Therefore, it is necessary to consider emotions when carrying out team related studies. Team passion is an important affective factor in teams. Passion refers to individual’s strong inclination to devote time and energy to activities that they like and think important (Vallerand et al., 2003a), which can be seen as an attitude based on affection. Permarupan et al. (2013) put forward that behaviors of team members can be evaluated by passions toward their given tasks and roles in the team. Therefore, team passion plays a crucial role in the team as a typical team emotional state. Some scholars explore the factors that affect team passion such as transformational leadership (Peng et al., 2020), shared leadership (Salas-Vallina et al., 2022), temporal leadership (Zhang et al., 2022), and empowering leadership (Hao et al., 2017). It can be seen that leader’s behavior is an impossible-to-ignore antecedent of team passion. A few other scholars attempt to investigate the impact of another common typical leader’s behavior—team leader’s CMSs on team passion. Yin et al. (2020a) prove that team leader’s CMSs have a direct effect on team passion. However, there is no research to explore the mechanism through which that team leader’s conflict management styles impact on team passion by now. Do team leader’s CMSs have indirect effects on team passion by some mediators besides the direct effect? This manuscript is trying to response to the question by examining the intrinsic mechanism between them from the emotional perspective.

On the basis of affective events theory, it considers that work environment characteristics or work events can trigger employees’ affective reactions thus leading to changes of employees’ work attitudes and behaviors, we can infer that team leader’s conflict management styles, as a team event, are likely to influence team passion—a kind of team members’ attitude, through the mediation of some team emotional reaction. Therefore, it is expected that conflict management styles significantly influence team passion. Positive team emotional climate is an important variable of affective reactions (Levecque et al., 2014). Some scholars demonstrated that leader’s behaviors helps to create a team emotional climate (Chiang et al., 2020; Kim et al., 2021; Saleh et al., 2022). Therefore, this manuscript intends to probe the mediating role of positive team emotional climate. Furthermore, team emotional intelligence (TEI) is a kind of emotional abilities for people to comprehend, manage, and make use of affective information (Salovey and Mayer, 1990). Barsade (2002) proposed that emotional contagion has great influence on individual attitudes and group processes. People who can better perceive other’s emotions have the ability to consider the influence of their behaviors on others (Ingram et al., 2017), then decides the ways to adjust or express his emotions and his emotional impact. Similarly, a team with high TEI is more likely to infect other members’ emotions due that they are able to better perceive, understand and predict other members’ emotions. Consequently, this study posits team emotional intelligence moderates the relationships between team leader’s conflict management styles and team emotional variables.

In conclusion, the purpose of this manuscript is to investigate the mediating mechanism of team leader’s conflict management style on team passion and to determine the boundary condition of these impacts with emotional perspective. The results of this paper can make the following contributions to existing studies of conflict management. On the one hand, this paper expands the research scope of impacts of conflict management styles. On the other hand, this manuscript investigates the mediating and moderating effects of emotional variables between CMSs and team passion, which broadens the study on the mechanisms of CMSs on team outcomes to a certain extent.

Structure of the other parts of the manuscript is as below. The second part discusses the relationships among team leader’s CMSs, positive team emotional climate, and team passion. Furthermore, this part also discusses how TEI plays a moderating role between CMSs and positive team emotional climate. The third part discusses the method of this manuscript, then is the results and the discussions of results. Finally, the fourth part presents theoretical and practical implications, and put forward research limitations and future research agenda.

## Literature review and hypothesis

The conflict management theory is mainly originated from Management Grid Theory proposed by Blake and Mouton, which the management model is divided into five different degrees based on two dimensions. Rahim (1983) summarizes five types of CMSs in work teams, i.e., integrating, dominating, avoiding, compromising, and obliging. Then, Thomas (2008) divides CMSs into five kinds: cooperation, competition, avoidance, compromise, and adaptation. However, some scholars question the five-factor classification in virtue of high correlations between different styles (Ross and DeWine, 1988). For example, both compromising and avoiding aim at reducing the differences between the two parties involved in the conflict through indirect and circuitous ways. Besides, collaborating/integrating and compromising concentrates on emphasizing the common interests of both parties so as to calm the contradiction. Tjosvold (1998) divides the conflict management styles into three types: cooperative, competitive, and avoidant.

Cooperative conflict management style embodies an attempt to integrate all people’s interests, which emphasize the openness of others’ viewpoints, considering the interests of both sides and seek a satisfactory solution. Competitive conflict management style only thinks about their own interest and its typical manifestation is that one party’s power dominates the other. Avoidant conflict management style is characterized by flickering words, usually adopting evasive and avoidant methods when facing conflicts. People who adopt this style are not concerned about the outcome of the conflict. Team leaders, as the core of teams, have the duty to organize team and solve problems by using the resources such as human, financial, and material in work teams. Hence, team leaders prefer positive conflict management styles including cooperative and competitive rather than negative ones such as avoidant. Based on this classification, many scholars choose the styles of cooperative and competitive to conduct related research (Somech et al., 2009; Shih and Susanto, 2010; Esbati and Korunka, 2021). Consequently, this manuscript selects cooperative and competitive conflict management styles for research.

### Team leader’s conflict management styles and positive team emotional climate

The existing literature on the impacts of CMSs is able to split into two categories. One focuses on the impacts on team climate and team performance. Some studies confirm that different conflict management styles have different significant impacts on work performance (Shih and Susanto, 2010; Tabassi et al., 2018; Wang et al., 2020). Others concern about the impacts on members’ attitudes and behaviors, such as job dissatisfaction, bullying behavior, voice behavior, trust, and emotional exhaustion (Erkutlu and Chafra, 2015; Leon-Perez et al., 2015; Way et al., 2016; Einarsen et al., 2018; Esbati and Korunka, 2021). As for the influences of leader’s conflict management styles, studies explore their impacts on team performance, employee behaviors, and team climate (Hempel et al., 2009; Erkutlu and Chafra, 2015; Tabassi et al., 2018) according to social cognitive theory, social exchange theory, and other related theories from the cognitive perspective. Chiang et al. (2020) believe that emotional factors play important roles in team activities. Thus, it is necessary to explore how team leader’s CMSs influence team emotional factors from the emotional rather than the cognitive perspective, so as to provide greater values of conflict management for team development. Scholars generally divide emotions into two dimensions, positive and negative emotions. Positive emotions serve as markers of flourishing, or optimal well-being and in peoples’ lives can be expressed as joy, interest, contentment, and love (Fredrickson, 2001). Negative emotions reduce individual pleasure, as shown in anxiety, sadness, anger, and despair (Fredrickson, 2001; Rank and Frese, 2008). When the internal consistency coefficient of emotion in the team is high, which indicates that the emotions of team members are consistent, team emotional climate can be considered to exist (George and Bettenhausen, 1990). Liu et al. (2008) propose that team emotional climate is a common perception of team members to moods and affective interaction in a team which characterizes a team and has a significant influence on teams and members.

As mentioned above, affective events theory emphasizes that work environment characteristics and work events can trigger emotional reactions of employees. Team leader’s behavior, as an event, can affect team members’ emotions and create some team emotional climate. Some researches suggest that leader’s behavior is essential to create team emotional climate (Chiang et al., 2020; Kim et al., 2021; Saleh et al., 2022). Eisenbeiss et al. (2008) discover that transformational leadership positively impact on team climate shaping. Chiang et al. (2020) declare that authoritarian leadership has a positive relationship with motion suppression climate. Therefore, as one of the significant manifestations of team leader’s behaviors, team leader’s conflict management styles should be able to influence team climate. When dealing with conflicts, team leaders who adopt the cooperative approach usually show respects for team members’ views and actively take a relatively fair way to communicate with team members so as to promote cooperation among the teams, which make team members feel relaxed, equal, free and happy, thus promoting a positive team emotional climate (Liu et al., 2008). Instead, team leaders who adopt the competitive approach tend to impose their opinions on team members. It will lead team members in a passive state which may make them produce negative emotions such as disappointment, tension, suspicion, and fear. It can be inferred that these negative emotions are possible to hinder communications among team members and even aggravate interpersonal relationships, resulting in the deterioration of positive team emotional climate. According to the analysis above, we put forward the following hypotheses:

*H1:* Team leader’s conflict management styles significantly affect positive team emotional climate.

*H1a:* Team leader’s cooperative conflict management style has a positive relationship with positive team emotional climate.

*H1b:* Team leader’s competitive conflict management style has a negative relationship with positive team emotional climate.

### Team leader’s conflict management styles and team passion

By now, most of current researches on the influences of conflict management styles are conducted from a cognitive perspective. As emotions have always influenced behaviors, it is valuable to probe the influence of CMSs on emotional factors at team level (Yin et al., 2020a). Team passion is a kind of emotional factor which plays a vital role in teams (Salas-Vallina et al., 2020, 2022; Mindeguia et al., 2021b). Based on self-determination theory, Vallerand et al. (2003b) define passion as an intensive inclination for people to invest time and effort toward an activity that they like and think is important and categorized it into both harmonious and obsessive passion. The meaning of harmonious passion is internalization by self-determination, which conducts people to join in an activity choicefully and freely. In comparison, obsessive passion comes from an interesting activity that is internalization by non-self-determined method. This kind of passion adjusts actions that are inconsistent with one’s self-concept (Vallerand et al., 2003b). This manuscript discusses about harmonious passion. Although passion is not clearly defined at the team level (Cardon et al., 2009), many scholars research the role of passion at the team level (Permarupan et al., 2013; Hao et al., 2017; Peng et al., 2020; Uy et al., 2021; Salas-Vallina et al., 2022; Zhang et al., 2022). Hence, this manuscript defines team passion as the degree with which a team experiences strong enthusiasm and investment to the team. Because work is indispensable in life, scholars have paid more attention to the passion in the workplace recently (Vallerand et al., 2003b; Zigarmi et al., 2009; Perttula and Cardon, 2012). One line of research has investigated how leaders influence employees’ passion. It is found that leader’s behavior significantly impacts staffs’ work passion, for example, ambidextrous leadership (Ma J.F. et al., 2018) and shared leadership (Salas-Vallina et al., 2022) positively influence members’ work passion. When team leaders adopt cooperative CMS, they usually encourage team members to actively share different viewpoints for discussion, deepen mutual understanding, and promote better cooperation between them. This can help members establish their values in the team and make them willing or even eager to benefit the team and the organization, thus enhancing the team passion. Whereas, team leaders adopt competitive conflict management style tend to impose their thoughts on members. Team members in a passive state may have negative emotions, which hinder the willingness of communication and even aggravate the degree of interpersonal conflicts, thus negatively affecting team passion. According to the analysis above, we propose the following hypotheses:

*H2:* Team leader’s conflict management styles have significant impacts on team passion.

*H2a:* Team leader’s cooperative conflict management style is positively related to team passion.

*H2b:* Team leader’s competitive conflict management style is negatively related to team passion.

### The mediating role of positive team emotional climate

The positive team emotional climate refers to the shared perception of the moods and affective interactions among team members as mentioned above (Liu et al., 2008). With positive team emotional climate, there is likely to create a positive emotional “infection” effect within teams, which can produce or maintain a positive emotional state within teams. Based on affective events theory, emotion is considered as a mediating mechanism in the event-outcome relationship. Team members develop shared attitudes, emotional responses, and behavioral patterns through shared experiences or events, and then it will have an influence on team attitudes and behaviors at a higher level conversely. Work passion requires strong positive emotions and internal driving forces and includes the emotional experience of strong work orientation, which leads employees to consider their work as an inner part and want to devote time and energy in it (Vallerand et al., 2003a; Birkeland and Nerstad, 2016). That is to say, team leader’s conflict management style, as a kind of leadership behavior, can be regarded as a common experience by team members, thus shaping team emotional climate and further stimulating team passion. Yin et al. (2020a) propose that team leader’s conflict management style positively affects positive team emotional climate. Zhu et al. (2022) also prove that paternalistic leadership is positively related to positive team emotional climate. In addition, Permarupan et al. (2013) confirm that the positive organizational climate is conducive to promoting employees’ work passion. Moreover, Mindeguia et al. (2021a) prove that positive climate is a mediating variable between transformational leadership and team passion.

Leaders who adopt a cooperative conflict management style are usually committed to achieving satisfactory results for both parties (Tjosvold et al., 2006). They try to integrate the interests of all members together to form a common best solution (Tjosvold et al., 2006; Desivilya et al., 2010). Then, they pay attention to members’ feedback in time and form a free and pleasant environment for communication, thus stimulating the positive emotions of team members and building a positive team emotional climate. Meanwhile, the positive emotional climate makes team members feel comfortable and motivates their passion. Therefore, leader’s cooperative CMS improves team passion by building a positive team emotional climate. Similarly, leader’s competitive CMS reduces team passion by inhibiting positive team emotional climate. Based on hypothesis 1, hypothesis 2, and the above illustration, we propose the following hypotheses:

*H3:* Positive team emotional climate mediates the relationships between team leader’s conflict management styles and team passion.

### The moderating effect of team emotional intelligence

The meaning of team emotional intelligence (TEI) is the competence of teams to formulate a series of standards to manage the processes of emotion (Lee and Wong, 2017). TEI is very important to improve the cooperation and cohesion of team members and promote the behavior of improving team efficiency (Lee and Wong, 2017). Wong and Law (2002) divide TEI as consisting of four dimensions: others’ emotion appraisal (OEA), self-emotion appraisal (SEA), regulation of emotion (ROE), and use of emotion (UOE). Among them, OEA is considered as the ability of individual to sense, comprehend and forecast other emotions. People who have such competence highly are more sensitive to others’ affections (Wong and Law, 2002). Ma and Liu (2019) propose that the impact of emotional intelligence in the context of conflict rests with OEA. Therefore, this manuscript adopts the dimension of OEA to represent and operationalize TEI. TEI is often considered as a method to solve challenging interpersonal relationships (Samiuddin et al., 2017). Miao et al. (2016) proved that emotional intelligence is possible to improve the degree of team members’ satisfaction. TEI can enhance the connection among coworkers, improve the quality of information exchange and decision-making, and reduce conflict in teams (Lee and Wong, 2017). Team with higher emotional intelligence can inspire the confidence and cooperation of team members, thereby building a positive and harmonious working atmosphere, thus promoting communication among members and reducing conflicts (Rego et al., 2007). Therefore, higher emotional intelligence is preferable to perceive the emotional fluctuations of team members in teams. A high TEI team can timely detect members with negative emotions and prompt them to revitalize themselves (Stubbs and Wolff, 2008), which can help create a more positive team emotional climate. We could inferred that high TEI is conducive to communication and conflict resolution in team, and thus improve the positive influence of cooperative CMS on positive team emotional climate and weaken the negative influence of competitive CMS on positive team emotional climate. Teams with low emotional intelligence may not easily recognize the tensions among members and the changes of members’ emotions, in which the barriers to communication between team members become more severe. Thus, low TEI could weaken the positive influence of cooperative CMS on positive team emotional climate and enhance the negative influence of competitive CMS on positive team emotional climate. Then the following hypotheses are proposed:

*H4:* Team emotional intelligence plays a moderating role between team leader’s conflict management styles and positive team emotional climate.

*H4a:* High team emotional intelligence enhances the positive influence of cooperative CMS on positive team emotional climate and weakens the negative influence of competitive CMS on positive team emotional climate.

*H4b:* Low team emotional intelligence weakens the positive influence of cooperative CMS on positive team emotional climate and enhances the negative influence of competitive CMS on positive team emotional climate.

Mindeguia et al. (2021b) propose that high emotional intelligence teams take on the role of “emotion manager” so as to create positive affective events for team members, which can arose passion of team members. Similar to the positive team emotional climate, high TEI teams could rapidly find out the members who lack passion for work and enhance their love for the team. By increasing less-passion members involvement in work, team passion could also be enhanced, which is conducive to the harmonious resolution of conflicts. Mindeguia et al. (2021b) put forward that there is a significant positive relationship between team emotional intelligence and passion. Therefore, this paper infers that teams with high TEI are sensitive to low-passionate members, while teams with low TEI are not. Consequently, high TEI could enhance the positive influence of cooperative CMS on team passion and weaken the negative influence of competitive CMS. On the contrary, low TEI could weaken the positive influence of cooperative CMS on team passion and enhance the negative influence of competitive CMS. Furthermore, Yin et al. (2020b) prove that the moderating role of TEI between team leader’s CMSs and team passion. Therefore, the following hypotheses are proposed.

*H5:* TEI has a moderating role between team leader’s conflict management styles and team passion.

*H5a:* High TEI enhances the positive influence of cooperative conflict management style on team passion and weakens the negative influence of competitive conflict management style on team passion.

*H5b:* Low TEI weakens the positive influence of cooperative conflict management style on team passion and enhances the negative influence of competitive conflict management style on team passion.

**Figure 1:**
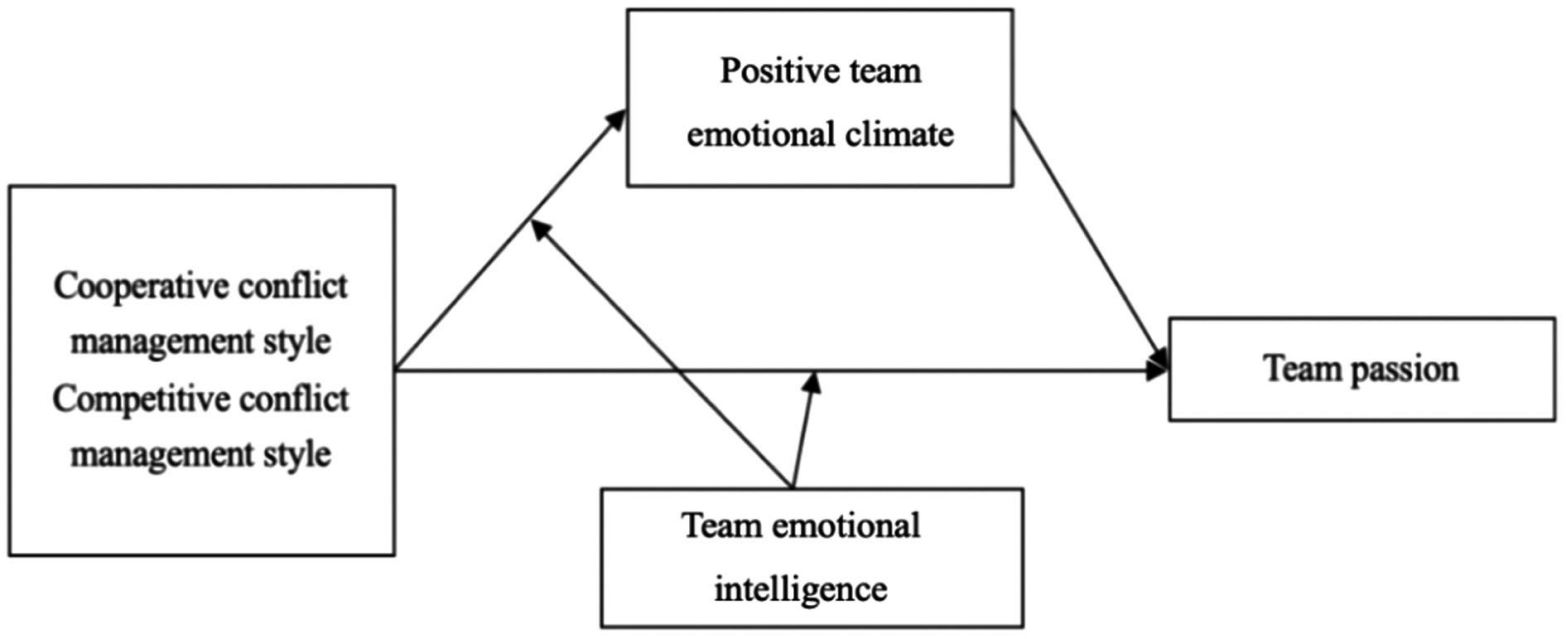
Conflict management styles on positive team emotional climate and team passion.

Therefore, the hypothesized model is displayed in Figure 1.

## Materials and methods

### Sample and data collection

In this paper, we conduct surveys to state-owned enterprises and internet enterprises in Beijing, Shenzhen, Shenyang, China, such as Baidu, Tencent, JD.COM, and Original Chemical by contacting relevant managers of these enterprises ahead of time and visiting the work places with the help of acquaintances and MBA alumni. This study collected team data in departments, which mainly work in a small group, such as sales, R&D, and propaganda, were chosen for the research. Because of the different size of work teams, each team surveyed a team leader and 3–6 team members. Finally, 484 valid surveys were collected, including 101 team leaders and 383 team members. We used anonymous questionnaire to ensure that it can truly reflect the respondents’ ideas. Questionnaires related to conflict management style were responded by leaders, while those related to team passion, positive team emotional climate and TEI were responded by team members. In order to match team leaders and members, we designed three items such as “what’s you last name” and “what’s your team leader’s last name.” For team leader, 86.79% respondents were males. On the aspect of age, 62.86% of the respondents was between 26 and 35 years, which is the most. The largest proportion held a bachelor degree, reaching 65.7%. After that, master degree or above reached 13.3%. The rest are below bachelor degree. In terms of working years, 50.48% respondents have been working for 6–10 years. For team members, 75.82% respondents were males. On the aspect of age, respondents between 26 and 35 years arrived 60.71%, which is the most. 84.9% respondents held a bachelor degree, then master degree or above reached 7.5%, the rest are below bachelor degree. 51.13% respondents have been working for 0–5 years.

### Measures

We selected internationally established scales and translated into Chinese to measure these variables. To ensure the equivalency of meaning, we translated questionnaire from English to Chinese and back to English. We adopted Likert five-point scale to measure the items of the questionnaire.

#### Conflict management styles

In this paper, we measured team leader’s cooperative and competitive conflict management styles by the scale established by Tjosvold et al. (2006). It measured cooperative and competitive conflict management style with nine items, which is answered by leaders. Specifically, cooperative conflict management style included five items such as “My work so that to the extent possible we all get what we really want” and competitive conflict management style contained four items such as “I demand that others agree to my position.”

#### Team passion

This study adopted the scale designed by Vallerand et al. (2003b). The scale had total seven items. For example, “This activity allows me to live a variety of experiences,” “This activity reflects the qualities I like about myself” and etc.

#### Positive team emotional climate

This study used three items of the positive team emotional climate scale derived from team emotional climate measure created by Liu et al. (2008). Example items included “In our team, members are optimistic and confident” and “Working in my team, members feel hopeful.”

#### Team emotional intelligence

As mentioned above, since the dimension of OEA reflects the vital role of TEI in the context of conflict, which can help people to perceive, understand, and predict other emotions (Ma and Liu, 2019). We adopted the items about OEA in the scale created by Wong and Law (2002). Example items included “I am sensitive to the feelings and emotions of others” and “I have good understanding of the emotions of people around me.”

#### Control variables

In this paper, the gender, age, education level, and working years of participants, which may have potential impacts on CMS, team climate, and team passion are taken as control variables (Rahim, 1983; Lewis, 2000; Beitler et al., 2018).

## Data analysis and results

In this manuscript, SPSS 24.0 and AMOS24.0 (Podsakoff et al., 2003) were used for descriptive statistical analysis, reliability analysis, and structural validity test. Mplus 8.0 was used both for the multiple regression analysis and hypotheses testing (MacKinnon et al., 2002; Muthén and Muthén, 2012). We adopted bootstrapping method to enhance the statistical effectiveness (Grant and Berry, 2011) by extracting 5,000 a^*^b values repeatedly from the raw data and confirm their unbiased interval.

Table 1 summarized the means, variances, correlation coefficients, and reliability test results for each major variable. According to Table 1, the Cronbach alpha values of all variables is over 0.7, thus each scale has good reliability.

**Table 1:** Descriptive statistical analysis and reliability.

	Mean	SD	1	2	3	4	5
1. Cooperative conflict management	3.36	0.38	**0.821**				
2. Cooperative conflict management	2.67	0.86	0.013	**0.811**			
3. Positive team emotional climate	3.47	0.4	0.794^**^	−0.036	**0.9**		
4. Team emotional intelligence	3.71	0.4	0.383^**^	0.253^*^	0.424^**^	**0.859**	
5. Team passion	3.57	0.41	0.751^**^	0.047	0.906^**^	0.597^**^	**0.948**

Bold value is Cronbach’s alpha. *N* = 101; ^**^p < 0.01.

### Structural validity

Confirmatory factor analysis was conducted in this study. We used Mplus8.0 to test the structural validity. Table 2 was the results.

**Table 2:** Structural validity test of different source scales.

**Level**	**Model**	**Factor**	***X***^**2**^	***df***	**GFI**	**CFI**	**NFI**	**RMSEA**
Team-member	Single-factor	PTEC + TEI + TP	383.58	54	0.85	0.92	0.9	0.13
	Two-factor A	PTEC + TEI,TP	267.6	53	0.89	0.95	0.93	0.1
	Two-factor B	PTEC,TEI + TP	273.19	53	0.91	0.95	0.94	0.1
	Two-factor C	PTEC + TP,TEI	267.6	53	0.89	0.95	0.93	0.1
	Three-factor	PTEC,TEI,TP	120.58	51	0.95	0.98	0.97	0.06
Team-leader	Single-factor	COO + COM	233.36	54	0.68	0.52	0.47	0.18
	Two-factor	COO, COM	83.61	53	0.9	0.92	0.86	0.07

*N* = 101, PTEC, positive team emotional climate; TEI, team emotional intelligence; TP, team passion; COO, cooperative conflict management style; and COM, competitive conflict management style.

At team-member level, the fitting degree of three-factor model is good (χ^2^/df = 2.36, GFI = 0.95, CFI = 0.98, NFI = 0.97, RMSEA = 0.06). And three-factor model is superior to single-factor model (combine PTEC, TEI, and TP) and two-factor models (combine PTEC, TEI, and TP in pairs). At team-leader level, the fitting degree of two-factor model is good (χ^2^/*df* = 1.58, GFI = 0.90, CFI = 0.92, NFI = 0.86, RMSEA = 0.07), which is superior to single-factor model.

### Data convergent testing at team level

In this paper, the consistency reliability ICC(1), ICC(2), and Rwg were calculated to test whether the data is able to converge at team level. According to the test, the ICC(1), ICC(2), and Rwg of team passion were 0.23, 0.47, and 0.893, separately. PTEC were 0.23, 0.48, and 0.893 separately. TEI were 0.28, 0.53, and 0.885 separately. Therefore, the above variables are able to converge at team level.

### Hypotheses test

In this manuscript, variance inflation factor (VIF) was tested by Model 1 to prevent multicollinearity. All VIF values were less than 5, illustrating that there was no multicollinearity problems. Refer to Table 3 for details.

**Table 3:** Hierarchical regression results.

**Variables**	**Model 1**	**Model 2**	**Model 3**	**Model 4**	**Model 5**	**Model 6**
	**TP**	**PTEC**	**TP**	**TP**	**TP**	**PTEC**
**Control variables:**						
Leaders’ gender	0.097 (1.207)	0.043	0.079	0.049	0.048	0.008
Leaders’ working years	0.151 (2.899)	−0.117	−0.007	0.096	0.087	−0.153
Leaders’ age	−0.084 (2.166)	−0.108	−0.151	−0.067	−0.071	−0.034
Leaders’ education level	0.004 (1.328)	0.021	0.035	0.008	0.004	−0.018
Team working years	−0.152 (4.263)	0.037	−0.045	−0.082	−0.073	0.012
Team average age	0.067 (3.085)	0.129	0.124	0.03	0.025	0.186
Team average education level	0.020 (1.534)	−0.008	0.064	0.091	0.088	−0.077
**Arguments:**						
COO	−0.043 (1.332)	0.785^***^	0.729^***^		0.069	0.773^***^
COM	0.062 (1.286)	−0.082	0.001			
**Mediating variable:**						
PTEC	0.773^***^ (2.266)			0.895^***^	0.842^***^	
**Moderating variable:**						
TEI	0.181^**^ (2.361)					0.153
**Interactive variable:**						
COO × TEI						−0.226^*^

*N* = 101, ^***^p < 0.001, ^*^p < 0.01. COO, cooperative conflict management style; COM, competitive conflict management style; PTEC, positive team emotional climate; TEI, team emotional intelligence; and TP, team passion.

#### Test of direct effects

As shown in Table 3, control variables were gender, working years, age, education level of team leaders and average working years, age, and education level of team members, the regression results of different independent variables to dependent variables were obtained, respectively. In model 2, cooperative and competitive conflict management styles were independent variables and positive team emotional climate were dependent variables. We found that the cooperative CMS positively influence positive team emotional climate ( β _1_ = 0.785, *p* < 0.001), hypothesis 1a was supported; while competitive CMS had no significant influence on the positive team emotional climate ( β _2_ = −0.082, *p* > 0.05), which not supported hypothesis 1b. In model 3, cooperative and competitive conflict management styles were independent variables and team passion was dependent variable. We found that cooperative CMS positively affected team passion ( β _3_ = 0.729, *p* < 0.001), hypothesis 2a was supported; however competitive CMS had no significant influence on team passion ( β _4_ = −0.001, *p* > 0.05), hypothesis 2b was not supported. We took positive team emotional climate was independent variable and team passion as dependent variable in model 4. Model 4 had a path coefficient β _5_ of 0.895 (*p* < 0.001), which illustrated that positive team emotional climate was significantly related to team passion, hypothesis 3 was initially validated. On the basis of above models, model 5 took positive team emotional climate as a mediating variable. Because the competitive style has no significant influence in models 2 and 3, it is unnecessary to keep on discussing the mediating impact of positive team emotional climate between competitive style and team passion and the moderating impact of team emotional intelligence between CMS and dependent variables. Consequently, model 5 did not contain competitive conflict management style. The path coefficient β _6_ between CMS and team passion was 0.069 (*p* > 0.05) and coefficient β _7_ was 0.842 (*p* < 0.001), which further supported hypothesis 3.

#### Test of indirect effects

Then, we used bootstrapping to exam indirect effects and confidence interval. According to the above, the path coefficients of independent variable-mediating variable ( β _1_) and mediating variable-dependent variable ( β _7_) were significant. Then repeated sampling 5,000 times, the mediating effect of positive team emotional climate was significant ( β = 0.258, *p* < 0.001, 95% CI of β _1_^*^ β _7_ is [0.583, 0.813], excluding 0).

**Figure 2:**
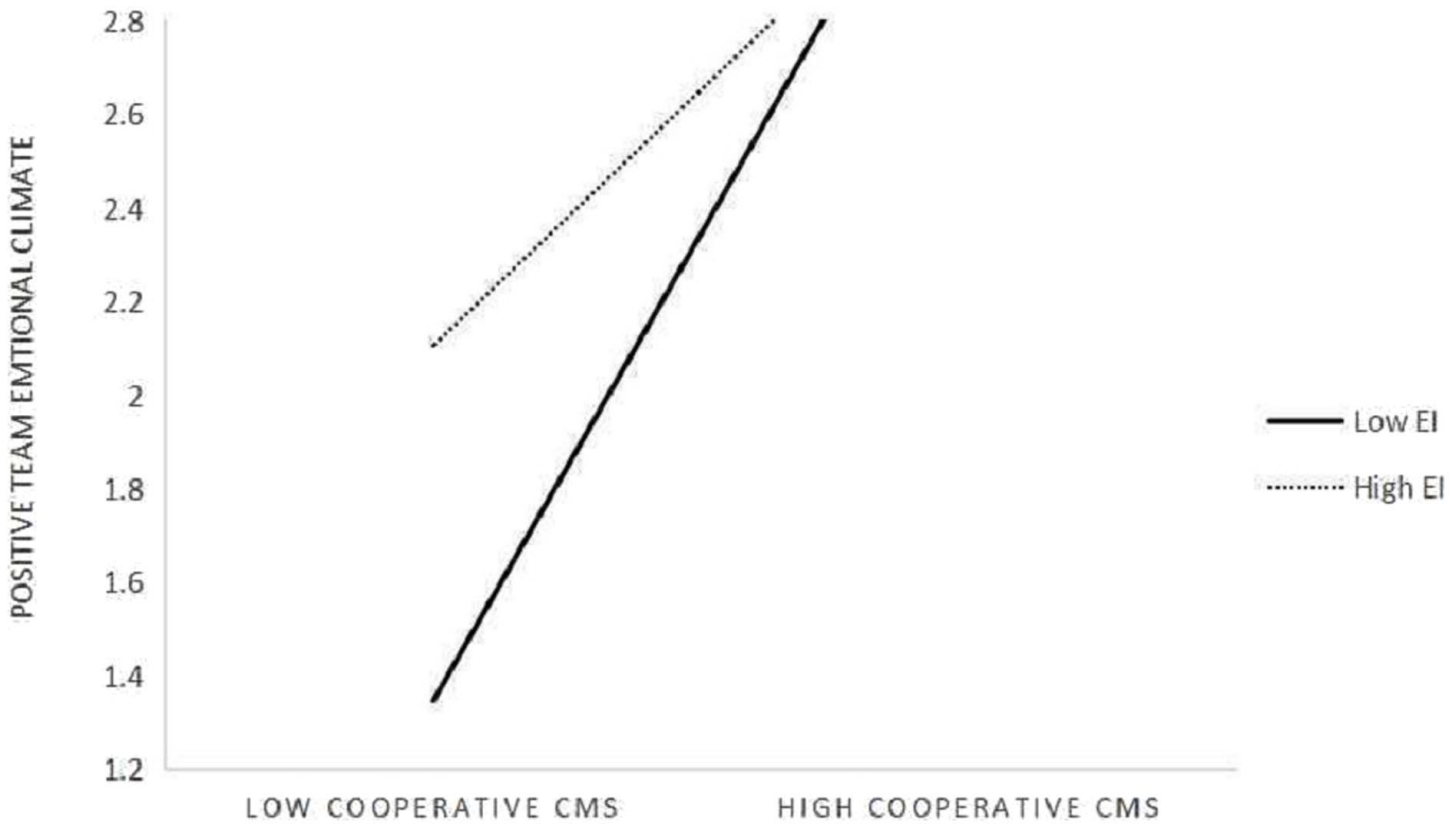
Cooperative styles × TEI influence on positive team emotional climate.

Finally, this manuscript investigated the moderating impact of TEI. Because positive team emotional climate completely mediated the relationship between cooperative CMS and team passion, it was not necessary to test its moderating role between them. Therefore, hypothesis 5 was not supported. The following analysis only examined its moderating effect between cooperative CMS and positive team emotional climate, and the results were shown in model 6. The path coefficients between interaction variable and positive team emotional climate was significant (β _8_ = −0.226, *p* < 0.01), which was opposite to hypotheses 4. In this study, method of Aiken and West (1991) was used to further describe team emotional intelligence’s moderating effect. The results are shown as Figure 2. According to Figure 2, we can find that whether in higher emotional intelligence teams or in lower emotional intelligence teams, leader’s cooperative CMS positively impact positive team emotional climate ( β _9_ = 0.671, *p* < 0.001; β _10_ = 0.921, *p* < 0.001). In addition, with team emotional intelligence higher, the positive effect of cooperative CMS on positive team emotional climate was weaker.

### Discussion

Table 4 showed that cooperative CMS was beneficial to creating a positive team emotional climate and improving team passion. This is because the cooperative CMS requires both parties involved to solve the conflict through open, cooperative, peaceful, and friendly methods. It can not only help create a happy and harmonious team climate, but also mobilize the working motivation of teams. The results also verified the positive impact of positive team emotional climate on team passion. For indirect effects, it showed that positive team emotional climate completely mediated the relationship between cooperative CMS and team passion. Moreover, team emotional intelligence played a moderating effect between cooperative CMS and positive team emotional climate.

**Table 4:** Hypotheses results.

Hypothesis	Results
1. Team leaders CMS on PTEC	Partly confirmed
1a. COO on PTEC	Confirmed
1b. COM on PTEC	Declined
2. Team leaders CMS on TP	Partly confirmed
2a. COO on TP	Confirmed
2b. COM on TP	Declined
3. the Mediating role of PTEC between COO and TP	Confirmed
4. the Moderating role of TEI between CMS and PTEC	Partly confirmed
5. the Moderating role of TEI between CMS and TP	Declined

CMS, conflict management styles; COO, cooperative conflict management style; COM, competitive conflict management style; PTEC, positive team emotional climate; TEI, team emotional intelligence; and TP, team passion.

Nevertheless, some hypotheses were not supported. First of all, the effects of competitive conflict management style on positive team emotional climate and team passion were not significant. This may be related to the fact that the survey data came from China. The power distance of Chinese society is large. Members who grew up under Chinese traditional education are used to obeying the orders of leaders, even though the current working environment encourages teamwork and reduces the power distance. China is a high power distance society (Ma Z. et al., 2018), and employees accustomed to the commanding work style of leaders. Employees think that leaders have the right to dominant and order them and accept the competitive conflict management style of leaders when confront conflicts. Therefore, team leader’s competitive CMS may not negatively affect team passion and team positive emotional climate. In addition, Chinese culture emphasizes “harmony is precious.” When confronted with conflict, team members are more concerned about escaping from the conflict situation as soon as possible. At that time, even if leaders adopt a competitive conflict management style which members do not like, it is acceptable for team members in order to escape from the conflict environment. We assume that team members’ negative emotions brought by team leader’s competitive conflict management styles and their positive emotions brought by escaping from conflicts may cancel each other out. Therefore, it can be speculated that team leader’s competitive CMS will not affect team members’ emotions. Secondly, TEI plays a negatively moderating role between cooperative CMS and team positive emotional climate. It is likely that team members with high emotional intelligence can regulate and infect other members within the team. According to emotional contagion theory, individuals can influence others or groups through emotional transmission (Barsade, 2002). Consequently, when the level of team emotional intelligence is high, even if team leaders adopt a highly cooperative CMS, positive team emotional climate does not necessarily increase a lot, but keeps a stable standard.

## Conclusion and implications

### Conclusion

With the perspective of emotion, this manuscript carries out the intrinsic mechanism of two typical conflict management styles on team passion and builds a corresponding theoretical model including the mediating impact of positive team emotional climate and the moderating influence of TEI. The model is tested empirically by investigating the paired data of 101 team leaders and 383 team members. We can find that cooperative CMS is conducive to creating a positive team emotional climate, which can effectively improve team passion. That is to say, positive team emotional climate plays the fully mediating effect between team leader’s cooperative CMS and team passion. Furthermore, TEI plays the moderating effect between leader’s cooperative CMS and positive team emotional climate.

### Theoretical implications

First of all, this manuscript explores the impacts of conflict management styles on team outcomes based on affective events theory with emotional perspective. Previous researches on the results of conflict management are mostly based on cognitive perspective and the outcome variables are mainly individual and team performance (Somech et al., 2009; Shih and Susanto, 2010; Tabassi et al., 2017). Some scholars take organizational identification (Erkutlu and Chafra, 2015), team coordination (Tabassi et al., 2018), and workplace bullying (Einarsen et al., 2018) as outcome variables, but pay less attention to the impacts on team attitudes related to emotions. This manuscript probes the impacts of CMS on an important team attitude related to emotional called team passion from the emotional perspective which not only provides a new perspective for follow-up research but also expands the research scope of impacts of conflict management styles.

Secondly, this study uses cross-level research method to verify the relationships between team leader’s styles of managing conflict and team emotional outcomes. In the past, when examining the impacts of team leader’s behavior on team’s emotion, most studies focus on the influences of leadership behaviors, such as spiritual leadership (Afsar et al., 2016), transformational leadership (Arnold, 2017), empowering leadership (Kim et al., 2018), and authentic leadership (Agote et al., 2016), while few researches concentrate on that of leader’s conflict management styles. Because conflict is inevitable in the team, we can infer that team leader’s CMS as one of the most important manifestations of his/her behaviors should have important effects on team results. Based on this idea, this manuscript explores the relationship between team leader’s CMSs and team emotional outcomes, so as to enrich the literature on the antecedents of team outcomes.

Lastly, this manuscript investigates the intrinsic mechanism of team leader’s CMSs on team passion, confirms the mediating effect of positive team emotional climate between them, and the moderating influence of TEI between CMSs and positive team emotional climate. Previous studies have focused on mediating or moderating effects of cognitive variables, such as team effectiveness (Cheng et al., 2012) and psychological safety (Erkutlu and Chafra, 2015). Since emotions is critical to help individuals comprehend the internal mechanism of team attitudes and behaviors, this manuscript explores the underlying mechanism of team leader’s CMSs on team passion from the emotional perspective and investigates the mediating and moderating effects of emotional variables. This, in turn, broadens the study on the mechanisms of CMSs on team outcomes to a certain extent.

### Managerial implications

There are three main managerial implications as well.

Firstly, team leader’s CMSs have a major effect on team climate and team attitude. When teams occur conflicts, team leaders should consider adopting cooperative conflict management style because it is conducive not only to good communication and information exchange within teams, but also to the creation of the positive emotional climate and inspiration of team passion, which helps find more effective solutions to conflicts, ease the relationship between two parties involved in conflict as soon as possible, reduce the loss caused by conflict, improve team performance and etc. Therefore, organizations should guide team leaders to emphasize and adopt cooperative conflict management style to cope with conflicts. Organizations can use case analysis, role-playing, mentor guidance, and other training methods to enhance team leaders’ skills of using cooperative conflict management style. In addition, organizations can also create a harmonious and cooperative working atmosphere that encourages leaders to adopt a cooperative conflict management style in the workplace, so as to improve team innovation performance.

Secondly, as employees’ emotions and working conditions are inseparable, employees’ emotional factors which cannot be ignored since they significantly affect the performance of enterprises or teams should be highly valued by managers. This paper proves that team emotional climate has a significant influence on team outcomes. In addition, leaders and managers should pay more attention to team passion which is the motivation for members to work hard. Therefore, team leaders should not only care about the management of team members’ personal emotions, but also emphasize the shaping of team emotional climate and the stimulation of team passion. On the one hand, it is suggested that team leaders create an open, relaxed, free and friendly atmosphere within the team through team building to arouse team passion. On the other hand, team leaders also can inspire team passion by setting clear and promising goals for team prospects.

Finally, researchers have already found the significance of emotional intelligence at work (Alkahtani, 2015; Lee and Wong, 2017; Jamshed and Majeed, 2018; Mindeguia et al., 2021b). The result of this study shows that TEI weakens the influence of cooperative conflict management style on positive team emotional climate. This shows that we do not want to promote the idea that members in teams with high emotional intelligence should be superior, because all members have emotion swings and even individuals with high emotional intelligence cannot avoid negative emotions (Jordan et al., 2002). Moreover, too high TEI is likely to inhibit the promotion and dissemination of a team’s positive emotions since teams with emotional intelligence at a high level may have a strong ability to influence team members’ emotions. Therefore, team leaders should not blindly pursue too high team emotional intelligence, but keep it on a relatively moderate level, so as to play its biggest role.

## Limitations and future research directions

Firstly, this manuscript investigates the intrinsic mechanism of team leader’s CMSs on team passion with the perspective of emotion and discovers the mediating effect of positive team emotional climate between them. Future researches can further probe the mediating effect of other emotional variables such as emotional control and affective tone (Rank and Frese, 2008). Secondly, this manuscript only chooses team leader’s CMSs of cooperation and competition. of course, leaders can also adopt other conflict management styles. Thus, future studies can further probe the influence of other CMSs on team outcomes based on emotional perspective. Thirdly, this study just chooses OEA dimension to measure team emotional intelligence. Although it makes sense to some extent, it is relatively simple and not necessarily able to stand criticizing. Consequently, future studies can add other dimensions to measure team emotional intelligence. Fourthly, the data in this manuscript totally came from questionnaire and are measured by self-report lacking of data from multiple evaluation sources. Future researches will consider combining various survey methods to obtain sample data and expand the survey subjects to reduce the impact of homologous data. Finally, we adopt the cross-sectional design in this manuscript. We can collect more longitudinal data in the future.

## Data availability statement

The raw data supporting the conclusions of this article will be made available by the authors, without undue reservation.

## Author contributions

JY contributed to the conceptual design of the study. JY, MQ and MJ contributed to the drafting of the article. GL and ML contributed to the data analyzes. All authors contributed to the article and approved the submitted version.

## Funding

The research was supported by Beijing Knowledge Management Institute (5212210983) and the National Natural Science Foundation of China (No. 72002016).

## Conflict of interest

The authors declare that the research was conducted in the absence of any commercial or financial relationships that could be construed as a potential conflict of interest.

## Publisher’s note

All claims expressed in this article are solely those of the authors and do not necessarily represent those of their affiliated organizations, or those of the publisher, the editors and the reviewers. Any product that may be evaluated in this article, or claim that may be made by its manufacturer, is not guaranteed or endorsed by the publisher.
